# Rapidly shifting maturation schedules following reduced commercial harvest in a freshwater fish

**DOI:** 10.1111/eva.12285

**Published:** 2015-07-16

**Authors:** Zachary S Feiner, Stephen C Chong, Carey T Knight, Thomas E Lauer, Michael V Thomas, Jeffrey T Tyson, Tomas O Höök

**Affiliations:** 1 Department of Forestry and Natural Resources, Purdue UniversityWest Lafayette, IN, USA; 2 Ontario Ministry of Natural ResourcesSault Ste. Marie, ON, Canada; 3 Division of Wildlife, Ohio Department of Natural Resources, Fairport Fish Research StationFairport Harbor, OH, USA; 4 Department of Biology, Ball State UniversityMuncie, IN, USA; 5 Michigan Department of Natural Resources, Lake St. Clair Fisheries Research StationHarrison Township, Mt. Clemons, MI, USA; 6 Division of Wildlife, Sandusky Fisheries Research Unit, Ohio Department of Natural ResourcesSandusky, OH, USA; 7 Illinois-Indiana Sea Grant, Purdue UniversityWest Lafayette, IN, USA

**Keywords:** Bayesian modeling, eco-evolutionary dynamics, fisheries management, fisheries-induced evolution, life history trait

## Abstract

Size-selective harvest of fish stocks can lead to maturation at smaller sizes and younger ages, which may depress stock productivity and recovery. Such changes in maturation may be very slow to reverse, even following complete fisheries closures. We evaluated temporal trends in maturation of five Great Lakes stocks of yellow perch (*Perca flavescens* Mitchill) using indices that attempt to disentangle plastic and evolutionary changes in maturation: age at 50% maturity and probabilistic maturation reaction norms (PMRNs). Four populations were fished commercially throughout the time series, while the Lake Michigan fishery was closed following a stock collapse. We documented rapid increases in PMRNs of the Lake Michigan stock coincident with the commercial fishery closure. Saginaw Bay and Lake Huron PMRNs also increased following reduced harvest, while Lake Erie populations were continuously fished and showed little change. The rapid response of maturation may have been enhanced by the short generation time of yellow perch and potential gene flow between northern and southern Lake Michigan, in addition to potential reverse adaptation following the fishing moratorium. These results suggest that some fish stocks may retain the ability to recover from fisheries-induced life history shifts following fishing moratoria.

## Introduction

Anthropogenic activity has the power to dramatically shift the strength and direction of selection on animal phenotypes (Darimont et al. 2009; Barbraud et al. 2013; Brown and Bomberger Brown 2013). Highly trait-selective harvest regimes, such as those exemplified by trophy hunting and commercial fisheries, may have the strongest influence on phenotypic change in exploited populations, acting through both plastic mechanisms driven by altered population densities and demographics and evolutionary change driven by selection for novel, and potentially maladaptive, phenotypes (Coltman et al. 2003; Walsh et al. 2006; Sutter et al. 2012). In particular, fisheries-induced shifts in life history traits represent an important issue facing many exploited fish stocks (Handford et al. 1977; Law 2000). Both empirical and modeling studies suggest that intense size-selective harvest selects for individuals that grow more slowly, mature at smaller sizes and younger ages, and increase reproductive investment (Conover and Munch 2002; Enberg et al. 2009; Nusslé et al. 2009; Sharpe and Hendry 2009). Such changes in life history traits can have dramatic influences on individual growth, mortality rates, offspring fitness, and population growth (Hutchings 2005), and thereby decrease stock productivity and yield (Hard et al. 2008) while increasing recruitment variability and sensitivity to environmental variation (Anderson et al. 2008; Hsieh et al. 2010).

Perhaps most troublesome, fisheries-induced changes in maturation and other life history traits appear to be very slow to reverse when fishing is reduced, potentially due to reduced genetic variation in exploited populations (Pinsky and Palumbi 2014) or lack of a strong selective pressure to restore historic phenotypes (Law and Grey 1989; Lahti et al. 2009). Experimental studies have yielded only partial recoveries of trait distributions after several generations (Conover et al. 2009; Salinas et al. 2012), and modeling simulations have shown that evolutionary changes to maturation schedules can take centuries to recover (Dunlop et al. 2009; Enberg et al. 2009; Kuparinen and Hutchings 2012). Therefore, the evolution of genetic traits related to maturation has been implicated in the lack of recovery of several overfished stocks following harvest reductions or fishery closures (Hutchings and Reynolds 2004).

The assessment of life history trait evolution can be complicated by plastic (i.e. nonevolutionary) responses to changes in growth or survival rates (Wang et al. 2008; Nusslé et al. 2009). Age at 50% maturity (A_50_) is a commonly used metric that represents the age at which 50% of the population is mature and can therefore detect whether fish are maturing earlier or later in life. However, A_50_ appears to be strongly influenced by plastic variation in maturation due to changes in growth or condition (Grift et al. 2003) and sampling-related biases (Wang et al. 2009b) and thus may have limited power to disentangle adaptive and plastic variation in maturation schedules. Probabilistic maturation reaction norms (PMRNs; Heino et al. 2002; Barot et al. 2004a) account for variation in growth and mortality by assessing the probability that a fish will first become mature at a given age and size and have been suggested as an improved indicator of evolutionary change in maturation schedules (Olsen et al. 2004; Dieckmann and Heino 2007) that are also robust to changes in sampling method, month, or agency (Wang et al. 2009b). Recent studies have observed close correlations between shifts in PMRNs and changing genotypic frequencies in exploited Atlantic cod (*Gadus morhua* Linnaeus) (Therkildsen et al. 2013) and demonstrated both phenotypic and genetic change in a suite of life history traits, including PMRNs under some conditions, after only five generations of selection in zebrafish (*Danio rerio* Hamilton) (Uusi-Heikkilä et al. 2015). However, caution should be used in interpreting differences in PMRNs as solely reflective of genetic or evolutionary factors because it remains unclear how well two-dimensional, length-based PMRNs truly discriminate between evolutionary and plastic changes in maturation schedules (Dieckmann and Heino 2007; Kraak 2007), as multidimensional PMRNs incorporating factors such as temperature or fish body condition have explained additional variation in individual maturation (Grift et al. 2007; Wright et al. 2011). Maturation may also be influenced by growth history, rather than absolute size, which could allow for plastic effects of growth to be reflected in PMRNs (Morita and Fukuwaka 2006). Therefore, we sought to evaluate the potential relative support for plastic or adaptive changes in maturation using both A_50_ and PMRNs, rather than overtly interpret shifts as solely indicative of plastic or evolutionary change.

Most of the research involving fisheries-induced evolution of life history traits in wild, commercially harvested stocks has focused on marine species (Sharpe and Hendry 2009). However, there is evidence of fisheries-induced evolution occurring in freshwater species as well (Handford et al. 1977; Edeline et al. 2007; Nusslé et al. 2009; Kokkonen et al. 2015). Yellow perch (*Perca flavescens* Mitchill) is an iteroparous, freshwater fish that supports the most valuable per unit mass commercial fishery in the Laurentian Great Lakes. Recently, many yellow perch stocks have experienced poor recruitment, steep population declines, and reduced commercial catch (Marsden and Robillard 2004). In southern Lake Michigan specifically, high exploitation rates significantly truncated age and size structures, skewed the sex ratio toward males, and caused a stock collapse in the mid-1990s (Wilberg et al. 2005; Lauer et al. 2008). In response, increasingly strict commercial and recreational fishing regulations were implemented beginning in the mid-1990s and the commercial fishery was closed in 1997 (Wilberg et al. 2005; Santucci et al. 2014). Similar reductions in stock size and shifts in sex ratios have occurred in other locations (e.g. Saginaw Bay and the main basin of Lake Huron; Fielder and Thomas 2006; Maurer et al. 2014), although commercial harvest has continued at varying levels for these and other stocks and yellow perch recovery has been slow (Kinnunen 2003; Baldwin et al. 2009). Comparing and contrasting temporal shifts in maturation among these stocks allowed us to evaluate how exploitation history may shape fish maturation schedules and observe whether maturation schedules are able to recover following harvest reductions or moratoria. We expected that commercial harvest would result in maturation at smaller sizes and younger ages and that recovery of those traits, if any, would be modest, even following the implementation of a fishing moratorium.

## Material and methods

Data were collected from five separate yellow perch stocks in the Great Lakes region: southern Lake Michigan; Saginaw Bay, Lake Huron; southeastern Lake Huron; and the western and central basins of Lake Erie. These populations represent different management units and have experienced differential exploitation histories from size-selective commercial (gill net and trap net) and recreational fishing (hook and line), which primarily selects for fish beyond approximately 200 mm total length (roughly age-3 individuals; Eshenroder 1977; Kinnunen 2003; Wilberg et al. 2005). The selectivity of the gill net fisheries is limited beyond about 300 mm (Wilberg et al. 2005), which corresponds to age-10 to age-12 fish, near the maximum ages commonly observed in the data (e.g. in Lake Michigan, age-12 and older fish comprised <0.5% of the total catch). Genetic evidence using microsatellite markers in multiple studies also suggests that these stocks are genetically distinct from each other using different metrics such as Bayesian structure analysis, amova, and indices of pairwise divergence (e.g. *F*_ST_, *R*_ST_, and *θ*_ST_; see Miller 2003; Parker et al. 2009; Sepulveda-Villet et al. 2011 for details). For each stock, data on individual fish sex, age, total length (mm), maturity status (mature or immature), and collection date were provided from annual surveys performed by collaborating agencies (Table1). All survey gears were standardized and implemented consistently over time (e.g. fished at the same sites using the same-sized meshes). Moreover, the highest age-specific catches (ages 2–4) were consistent over time for each stock and included the ages during which most individuals matured (i.e. large number of both immature and mature individuals), thus giving us the most certainty in our estimates of maturity for these ages (Barot et al. 2004a).

**Table 1:** Data-contributing agency, gear type, sample months, cohorts, and sample size (N) for male and female yellow perch maturation data included in this study. Agencies include Ball State University (BSU), Michigan Department of Natural Resources (MI DNR), Ontario Ministry of Natural Resources (OMNR), and Ohio Department of Natural Resources (OH DNR)

Population	Agency	Gear	Months	Cohorts	Female N	Male N
Lake Michigan	BSU	Trawl	June–August	1979–2006	4509	2891
Central Erie	OH DNR	Trawl	July–November	1982–2008	15 209	15 987
Western Erie	OH DNR	Trawl	August–October	1975–2010	4692	6738
Lake Huron	OMNR	Gill net	June–October	1972–2008	7703	11 274
Saginaw Bay	MI DNR	Trawl	September–October	1967–2004	6611	6897

For temporal analysis, each fish was assigned to the cohort corresponding to its year of birth. As fish were sampled, assessed for maturity, and aged during the summer and fall and yellow perch only spawn in the spring, the age of each fish was increased by one to reflect that it would not spawn until the beginning of the next year of life. Because the analytical methods used (described below) required large sample sizes, and to simplify comparisons of trends in maturation among stocks (because other ecological events such as species invasions and changes in water quality were not consistent or coincident among populations), cohorts were grouped by decade of birth for temporal analysis. Sex-specific A_50_ for each stock and decade was estimated using a hierarchical Bayesian logistic regression with maturity status as a binary response variable (1 = mature, 0 = immature) and a fixed effect of stock, random effect of decade within stock, and continuous effect of age as explanatory variables. The A_50_ for each stock and decade was estimated as the negative intercept divided by the slope (−*α*/*β*) of the regression and represented the age at which 50% of the population is mature. As an indicator of juvenile growth, Bayesian posterior estimates of the sex-, stock-, and decade-specific mean lengths at age 2 were also determined. Credible intervals for both A_50_ and mean length were defined as the 2.5th and 97.5th percentiles of 1000 drawn posterior estimates. To more closely examine the importance of growth to plastic changes in maturation from the 1980s through the 2000s, where data from all five populations were available, a linear regression with A_50_ as the dependent variable, stock and decade as factors, and the mean length at age 2 as a continuous covariate was also performed on each of 1000 posterior draws of the maturation and length models, yielding posterior estimates of the effects of each explanatory variable on maturation for each sex. Because A_50_ could not be estimated for all stocks and decades for male yellow perch (see Results), only data from Lake Michigan, central Lake Erie, and western Lake Erie were included in the male analysis, while information from all stocks was used in the female analysis.

Sex-, stock-, age-, and decade-specific PMRNs were evaluated using the Bayesian framework described by Wright et al. (2011), and the midpoints of each PMRN, that is, the age-specific (*a*) length at which a fish has a 50% probability of becoming mature, termed the Lp_50,a_, were used to detect adaptive differences over time. This method assumes that immature and mature individuals exhibit the same growth and mortality rates, which is likely violated in yellow perch. However, a sensitivity analysis concluded that PMRN analysis was robust to moderate violations of these assumptions, especially with sufficient sample sizes (Barot et al. 2004a); by grouping the data into decadal cohorts, sample sizes in this study were sufficient to provide robust estimates of PMRNs (Table1). First, a von Bertalanffy growth model was fit to each stock-, sex-, age-, and decade-specific dataset to determine the average growth increment between specific ages (*Δs*_a,a−1_). Next, the age-specific probability of a fish being mature at a given length (termed the maturation ogive, *o*_a,s_) was determined using logistic regression of length on binary maturation status for each age within a sex, stock, and decade. Finally, the probability that a fish had first matured (*m*_a,s_) was calculated as *m*_a,s_* = *(*o*_a,s_* – o*_a−1,s−Δs_)/(1 – *o*_a−1,s−Δs_) using 1000 random draws from the posterior estimates of each *Δs*_a,a−1_ and *o*_a,s_ (Barot et al. 2004a). Each sex-, stock-, decade-, and age-specific Lp_50,a_ was then determined by dividing the negative intercept by the slope (−*α*/*β*) of a logistic regression with *m*_a,s_ as the response and length as the explanatory variable (see Wright et al. 2011 for more details).

Calculation of each Lp_50,a_, A_50_, and mean length at age 2 were conducted using JAGS in R with package ‘rjags’ (Plummer 2003, 2013; R Core Team 2012). The initial 5000 steps were discarded to eliminate the influence of initial values, and an additional 5000 iterations with a thin rate of five (i.e. every fifth sample) were kept to define the mean, median, and 95% credible intervals of each respective mean length, A_50_, and Lp_50,a_. Four chains were used for each run, and convergence was confirmed via visual inspection of trace plots and Brooks–Gelman–Rubin convergence statistics near one (Brooks and Gelman 1998). Noninformative priors were used for all models (Table2). Temporal differences in maturation were identified via nonoverlapping credible intervals of estimates between decades (Wright et al. 2011). To directly compare rates of change in *Lp*_*50*_ estimates with those reported in other studies, interdecadal differences were converted to haldanes (*h*), expressed as change in number of standard deviations per generation (Gingerich 1993; Hendry and Kinnison 1999) as *h *= ([*x*_1_ – *x*_0_]/*s*_p_)/*g*, where *x*_1_ and *x*_0_ are the starting and end points of the phenotypic trait (*Lp*_50,a_), *s*_*p*_ is the pooled standard deviation of the respective *Lp*_50,a_ estimates for each population, and *g* is number of generations. Generation time was estimated using equation 4 in Devine et al. (2012) using a standard length–mass relationship to predict age-specific mass (as mass data were unavailable for most populations; Willis et al. 1991). *Lp*_50,a_-specific *s*_p_ was estimated following equation 3 in Devine et al. (2012). Estimates of *h* were averaged across ages to evaluate overall change in maturation schedules between time periods.

**Table 2:** Prior parameterizations used to develop models for age at 50% maturity, mean length at age 2, von Bertalanffy growth curves, and age-specific maturation ogives. Subscripts *i*,*j*, and *k*denote lake-, cohort-, or age-specific parameter values, respectively. In JAGS, normal distribution is specified using a mean (*μ*) and precision (*τ*), which is 1/variance. Gamma distributions (*Γ*) are defined using shape (*α*) and rate (*β*) parameters

Parameter	Definition	Prior definition	Prior
Age at 50% maturation model			
*α*_*i*_	Hyperprior for random intercepts	N(*μ*,*τ*)	N(0, 0.0001)
*β*_*i*_	Hyperprior for random slopes	N(*μ*,*τ*)	N(0, 0.0001)
*σ*_*α,i*_	Lake-specific standard deviation of intercept	U(min, max)	U(0, 10)
*σ*_*β,i*_	Lake-specific standard deviation of slope	U(min, max)	U(0, 10)
*α*_*i,j*_	Intercept of logistic regression	N(*μ*,*τ*)	N(*α*_*i*_, *σ*_*α,i*_)
*β*_*i,j*_	Slope of logistic regression	N(*μ*,*τ*)	N(*β*_*i*_, *σ*_*β,i*_)
*μ*_length,*i,j,k*_	Mean length of fish each lake, cohort, and age	N(*μ*,*τ*)	N(0, 0.0001)
*σ*_length_	Standard deviation of mean length	Γ(*α*,*β*)	Γ (0.001, 0.001)
von Bertalanffy growth model			
*L*_*∞i,j*_	Asymptotic length	N(*μ*,*τ*)	N(300, 0.001)
*K*_*i,j*_	Growth rate	Γ(*α*,*β*)	Γ (0.01, 0.01)
*t*_0*i,j*_	Predicted age where length is zero	U(min, max)	U(−2, 2)
*σ*	Common standard deviation	Γ(*α*,*β*)	Γ (0.01, 0.01)
Maturation ogive model			
*α*_*i,j,k*_	Intercept of logistic regression	N(*μ*,*τ*)	N(0, 0.0001)
*β*_*i,j,k*_	Slope of logistic regression	N(*μ*,*τ*)	N(0, 0.0001)

To evaluate how maturation schedules may have changed in response to fluctuations in harvest levels, the total annual harvest (tonnes) of yellow perch from each population was retrieved from Baldwin et al. (2009). Total commercial harvest is an imperfect indicator of exploitation pressure as it does not take into account changes in population abundance or fishing effort like other metrics, such as instantaneous mortality or exploitation rate. However, these metrics were not consistently available for all populations and time periods, so more readily available commercial harvest totals were used as a qualitative proxy.

## Results

**Figure 1:**
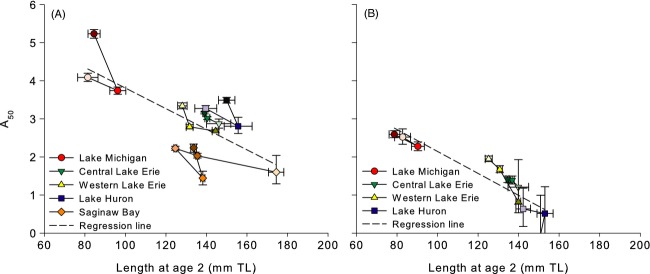
Patterns of (A) female and (B) male age at 50% maturity (A_50_) versus mean length at age 2 for yellow perch from Lake Michigan, central Lake Erie, western Lake Erie, Lake Huron, and Saginaw Bay (different colors and symbols) from 1980 to 2000 (Michigan, Erie, and Huron) or 1960 to 2000 (Saginaw Bay). Symbols are increasingly shaded from oldest (lightest; 1980 or 1960, respectively) to most recent (darkest; 2000) decadal observation and solid lines link symbols in temporal sequence. Error bars represent 95% credible intervals of each A_50_ or mean length estimate. Dashed line represents global regression line. Note that the regression for male A_50_ (panel B) included data from Lake Michigan, central Lake Erie, and western Lake Erie only, as estimates from Lake Huron and Saginaw Bay were limited to fewer decades because of data limitations. Lake Huron estimates are shown for illustration in panel B.

Age at 50% maturity significantly varied among stocks in both sexes and also among decades in females. In Lake Michigan and Lake Huron, temporal changes in female A_50_ followed a fluctuating pattern, decreasing from the 1980s to the 1990s, and increasing again from the 1990s to the 2000s by ∼1.5 years in Lake Michigan and ∼0.5 years in Lake Huron. The basins of Lake Erie and Saginaw Bay displayed differing trends; western Lake Erie declined through time by ∼0.5 years, while Saginaw Bay increased by ∼1 year and central Erie exhibited relatively little change. Male A_50_ exhibited similar patterns, but a reduced magnitude of change (0.3–1.0 years). Variation in A_50_ was strongly negatively associated with juvenile growth rate (size at age 2) in both sexes. Differences among stocks explained an average of 80% of the variation in A_50_ in females and 72% in males. Decade explained much less variation, only 8% and 5% of the total for each sex, respectively. Mean length at age 2 accounted for 5% of the variation in female A_50_ (or 46% of the remaining variation after accounting for stock and decade differences; Fig.1A) and 20% of the variation in male A_50_ (or 88% of the remaining variation after accounting for stock and decade differences; Fig.1B), and the credible intervals for both slopes relating A_50_ to mean length at age 2 did not include zero (females: mean = −0.04 mm TL, lower and upper limits of 95% CI = −0.06, −0.03; males: mean = −0.06 mm TL, lower and upper limits of 95% CI = −0.09, −0.04). Between sexes, males always matured at younger ages than females (Fig.1).

**Figure 2:**
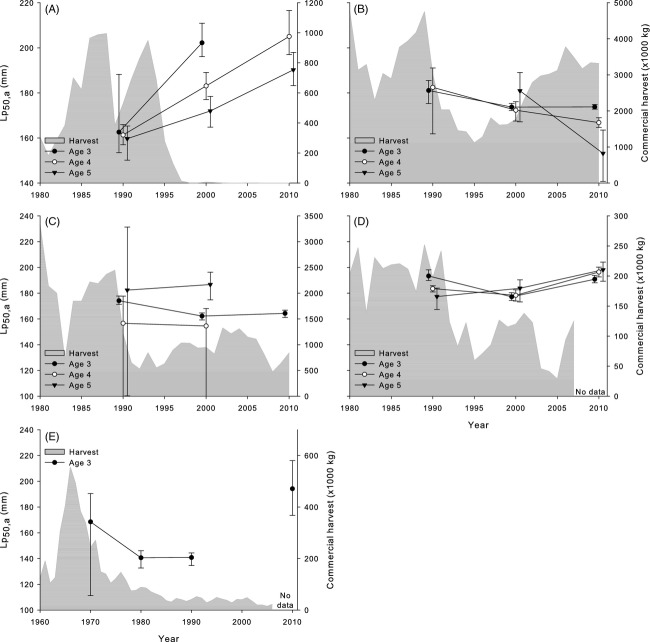
Total commercial harvest (×1000 kg; filled gray area) and temporal trends in the midpoints of PMRNs (Lp_50,a_; points and lines) for different ages of female yellow perch in (A) Lake Michigan, (B) central Lake Erie, (C) western Lake Erie, (D) Lake Huron, and (E) Saginaw Bay. Points for PMRNs are placed at the end of their respective decade (e.g. points corresponding to data from 1980 to 1989 are placed at 1990). Points for different ages are offset on the *x*-axis by 0.5 years for clarity. Note different secondary *y*-axis scales for commercial catch for each plot. The Lake Michigan commercial fishery was closed in 1997, and commercial harvest data for Lake Huron and Saginaw Bay were only available through 2007 and 2006, respectively. Commercial harvest data taken from Yellow Perch Task Group (YPTG) (1989, 1994, 2006, 2014), and Baldwin et al. (2009).

**Figure 3:**
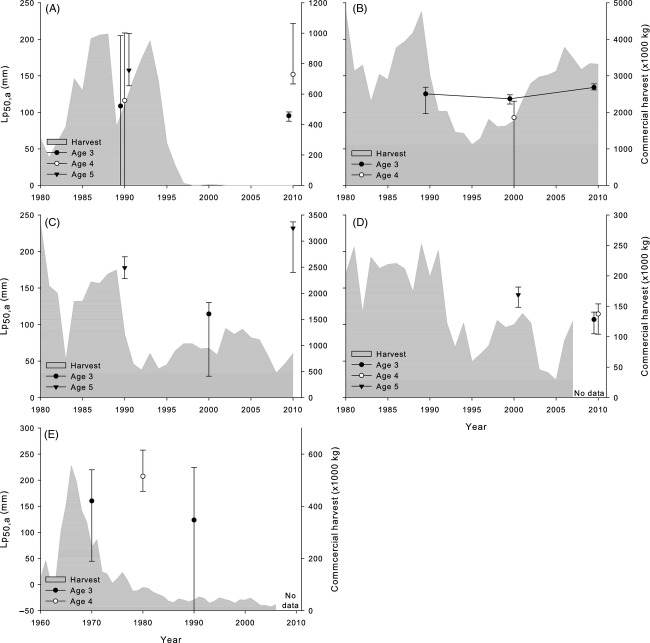
Total commercial harvest (×1000 kg; filled gray area) and temporal trends in the midpoints of PMRNs (Lp_50,a_; points and lines) for different ages of male yellow perch in (A) Lake Michigan, (B) central Lake Erie, (C) western Lake Erie, (D) Lake Huron, and (E) Saginaw Bay. Points for PMRNs are placed at the end of their respective decade (e.g. points corresponding to data from 1980 to 1989 are placed at 1990). Points for different ages are offset on the *x*-axis by 0.5 years for clarity. Note different secondary *y*-axis scales for commercial harvest for each plot. The Lake Michigan commercial fishery was closed in 1997, and commercial harvest data for Lake Huron and Saginaw Bay were only available through 2007 and 2006, respectively. Commercial harvest data taken from Yellow Perch Task Group (YPTG) (1989, 1994, 2006, 2014), and Baldwin et al. (2009).

In contrast to fluctuating patterns in A_50_, there were strong directional shifts in Lp_50,a_ estimates in three of the five yellow perch stocks which appeared to follow trends in the commercial harvests of each stock (Fig.2). Most strikingly, Lp_50,a_s for female yellow perch in Lake Michigan rapidly increased from the 1980s to 1990s (mean *h *=* *1.20) and continued to increase into the 2000s (mean *h *=* *0.86; Fig.2A). Saginaw Bay females followed a similar trend; after decreasing from the 1960s to the 1970s and 1980s (mean *h*from 1960s to 1980s = −0.47), Lp_50,a_s in the 2000s had increased even beyond their previous levels (mean *h*from 1980s to 2000s = 1.78; Fig.2E). Lake Huron females also exhibited a smaller temporal increase concomitant with a smaller decline in the harvest from that stock (mean *h*from 1980s to 2000s = 0.45; Fig.2D). In contrast, the Lake Erie stocks have sustained large harvests over time relative to the other populations, and female Lp_50,a_s declined in both the central basin (mean *h *=* *−1.18; Fig.2B) and western basin (mean *h *=* *−0.58; Fig.2C). Trends in male Lp_50,a_s were less conclusive. Due to generally young maturation leading to fewer immature males being captured, Lp_50,a_ was reliably estimated for fewer ages and time periods. For stocks where age-specific temporal comparisons were possible, male Lp_50,a_s had not changed over time (i.e. credible intervals overlapped; Fig.3) and rates of change were relatively slower (Lake Michigan mean *h *=* *0.07; central Lake Erie *h *=* *0.54, western Lake Erie *h *=* *0.38).

## Discussion

This study has documented rapid recovery of maturation schedules following reduced commercial harvest in a wild, exploited freshwater fish species. Yellow perch stocks that experienced a reduction in commercial harvest over time exhibited marked increases in Lp_50,a_ estimates of up to 50–60 mm in Lake Michigan and Saginaw Bay, corresponding to changes of 0.8 to 1.8 haldanes. Meanwhile, yellow perch in western and central Lake Erie were exposed to continuously high harvest and exhibited either no change or a decline in PMRNs over time. The directions of these responses would seem to fit the expected trajectories of shifts in maturation to changes in harvest rates predicted by both modeling studies and laboratory experiments (Kuparinen and Merilä 2007; Conover et al. 2009) and suggest that, while fisheries harvest can shape the life histories of exploited species, fish stocks could potentially retain the ability to recover trait values if fishing is reduced or ceased.

Although the direction of changes in maturation matched theoretical expectations, the magnitude of positive shifts in PMRNs observed in this study was large (nearly 2 haldanes for Saginaw Bay female yellow perch), especially considering that evidence suggests adaptation of maturation and other life history traits should be slow (Enberg et al. 2009). Devine et al. (2012) reported rates of change in PMRN estimates ranging from −2.2 to 0.9 haldanes across 23 stocks of exploited marine species, and Sharpe and Hendry (2009) found that most reported rates of phenotypic change in PMRNs were negative (8 of 11 exploited stocks). The true ability of PMRNs to disentangle variation in maturation due to plastic and genetic processes remains debated (Dieckmann and Heino 2007), thus some of this change could be due to phenotypic plasticity or adaptation not related to fisheries selection. Therefore, other potential explanations must be considered.

First, fishing is not the only stressor on Great Lakes yellow perch, as the region has suffered multiple species invasions (e.g. Bunnell et al. 2009), changes in water productivity and clarity (Bunnell et al. 2014), and fluctuations in yellow perch recruitment and abundance (e.g. Irwin et al. 2009; Ivan et al. 2011). In Saginaw Bay, poor water quality led to a reduced forage base for yellow perch, reducing growth and increasing mortality rates of adults even as fishing pressure was reduced in the 1970s and 1980s. Only after growth and habitat conditions improved in the 1990s did PMRNs increase. An increase in size-selective mortality on small, slow-growing individuals through walleye predation and poor overwinter survival may have also played a role in driving the observed increase in PMRNs in the 2000s (Ivan et al. 2011). Growth rates also increased in Lake Michigan from the 1980s to 1990s and may have some role in the large increase in PMRNs we observed between those decades, as more rapid growth rates could have led to larger age-specific sizes at maturation that were not accounted for by the PMRNs (Morita and Fukuwaka 2006). Large fluctuations in growth rates may also affect the adaptation of maturation schedules through life history trade-offs; high growth rates may favor maturation at large sizes, with large individuals becoming mature while smaller individuals remain immature and experience faster growth rates (Folkvord et al. 2014). However, growth and abundance of yellow perch in Lake Michigan declined from the 1990s to 2000s (Makauskas and Clapp 2010; this study) while PMRNs continued to increase, suggesting that adaptation in response to the restriction of harvest likely also contributed to the shifts in maturation schedules. Moreover, PMRNs in the western and central basins of Lake Erie changed very little over time despite experiencing similar ecosystem stressors as Lake Michigan and Huron in the form of species invasions, reduced nutrient loading, and large changes in yellow perch abundance (Bunnell et al. 2014; Yellow Perch Task Group (YPTG) 2014). Therefore, fishing pressure may be a general selective force shaping the maturation schedules of yellow perch stocks throughout the Great Lakes, even as other factors may influence local variation in maturation schedules.

Age at 50% maturity was particularly responsive to changes in growth rates, demonstrating the potential for the observed shifts in maturation to be confounded with changes in growth. Specifically, A_50_ was strongly negatively linked to mean length at age 2, indicating that decades with improved growth for juvenile fish tended to lead to maturation at younger ages. Alternatively, larger, more likely mature individuals may be more susceptible to capture in size-selective gears than smaller immature individuals, especially at younger ages, which may contribute to this pattern. However, a trade-off between maturation age and growth rate is well supported by life history theory (Stearns and Koella 1986) and has been observed in many fish species (Stearns and Koella 1986; Grift et al. 2003), supporting our contention that faster growth rates are also driving younger maturation ages. In some cases, this meant that shifts in A_50_ appeared to run counter to changes in PMRNs. For example, A_50_ declined in Lake Michigan from the 1980s to the 1990s with increases in length at age 2, while PMRNs increased during that time. This may reflect an increase in growth both promoting maturation at earlier ages and increasing size at maturation within each age class, as discussed above. That changes in A_50_ and PMRNs can appear to oppose one another (declining age at maturation with increasing PMRNs), suggests that both metrics should be considered when investigating temporal and spatial variation in maturation schedules, as plasticity due to changes in growth rates could potentially mask underlying dynamics in exploited fish stocks.

Second, rapid changes in life history traits due to changes in harvest practices in other studies have been linked to the immigration of novel genotypes (Pukk et al. 2013). Studies of genetic structure (Miller 2003), larval dispersal (Beletsky et al. 2007), and adult movement (Glover et al. 2008) of yellow perch in Lake Michigan suggest that the entire main basin of the lake represents a partially mixed stock distinct from either Green Bay (the largest bay of Lake Michigan; Kapuscinski and Miller 2000; Miller 2003) or Lake Huron (Parker et al. 2009). Because the most intense harvest existed primarily in the southernmost extent of Lake Michigan, it is possible that variable dispersal of larvae or adult yellow perch between northern and southern Lake Michigan resulted in the northern area serving as a reserve of genetic variability that enabled a more rapid recovery of maturation schedules once the strong selective pressure of commercial harvest was removed. Drowned river mouth lakes (i.e. lakes formed at the outlets of rivers into the main basin) are also common in Lake Michigan, contain yellow perch subpopulations, and may act as additional sources of genetic material distinct from the main lake body (Parker et al. 2009). Other heavily exploited fish populations have suffered from reduced genetic diversity, potentially reducing their adaptive potential (Hauser et al. 2002; Hutchinson et al. 2003). Small movements of even a few individuals, and their provision of new genetic material, have resulted in improved fitness and recovery of populations both plant and animal taxa by mitigating inbreeding depression (Richards 2000; Vilà et al. 2003) and increasing genetic diversity (Hutchinson et al. 2003; see also review by Whiteley et al. 2015), suggesting a similar mechanism could also act to speed the recovery of life history traits in exploited fish populations. Therefore, the stock structure and localization of fishing pressure in Lake Michigan may have ultimately influenced ability of the southern Lake Michigan population to recover following the commercial moratorium.

As suggested above, some studies question the ability of PMRNs to strictly distinguish between evolutionary and plastic changes in maturations schedules (e.g. Morita and Fukuwaka 2006; Uusi-Heikkilä et al. 2011). Even if the changes observed here are due more to phenotypic plasticity than evolutionary responses, relative differences in the levels of adaptive phenotypic plasticity among populations may moderate how each responds to fishing pressure. Hidalgo et al. (2014) found that increased levels of adaptive plasticity in populations could dampen the effect of fisheries exploitation on population dynamics, thereby altering how they phenotypically respond to fishing. Rapid phenotypically plastic shifts could theoretically improve stock resiliency and allow a faster recovery of previous life history phenotypes following reduced fishing pressure or improved growth conditions in some populations. Yellow perch have demonstrated significant plasticity in growth rates, morphology, and tolerance of stressors (Heath and Roff 1987; Svanbäck and Eklöv 2006; Lippert et al. 2007; Roberts et al. 2011), and there is also evidence that populations may vary in their respective levels of plasticity and genetic divergence in these traits (Victoria et al. 1992; Parker et al. 2009). Thus, populations that exhibit higher levels of plasticity than others may exhibit more rapid shifts in life history traits when encountering new environmental conditions or changes in fishing pressures, which could potentially have led to similar shifts in life history strategy as an adaptive response to changes in fishing pressure.

Finally, it is important to note that evaluating the effects of size-selective fisheries through total commercial harvest data may not represent a complete picture of the complex dynamics influencing life history trait expression. However, where measures of mortality or exploitation rates were available in the literature, their temporal patterns largely matched the observed trends in commercial harvest. In Lake Michigan, a recent stock assessment estimated yellow perch annual mortality rates as 63–80% in the 1980s and early 1990s and declining to about 33% annual mortality during the mid to late 1990s as regulations were instated (Wilberg et al. 2005). In the western basin of Lake Erie, mortality rates for yellow perch older than age-2 were roughly stable (although variable) around 50–60% throughout the time period (Yellow Perch Task Group (YPTG) 2014). In the central basin of Lake Erie, mortality and exploitation rates generally decreased through the 1980s and 1990s, but remained stable at about 40–50% annual mortality in the 2000s and always surpassed yellow perch mortality in Lake Michigan after the moratorium (Yellow Perch Task Group (YPTG) 2014). Finally, extremely high fishing pressure and poor growth conditions were attributed to the decline of the Saginaw Bay yellow perch population from the 1960s through 1980s (Eshenroder 1977; Schaeffer et al. 2000). More recently, commercial catch per unit effort and annual mortality rates have stabilized at 46–53% since 1986 (Fielder and Thomas 2006).

Despite these caveats, observed shifts in PMRNs may be at least partly representative of adaptive change for several reasons. As mentioned previously, changes in length-based PMRNs account for most of the variation in maturation due to the plastic effects of growth in length (Grift et al. 2007; Mollet et al. 2007), and shifts in PMRNs have been correlated with genetic change in other exploited species (Therkildsen et al. 2013). In another percid, pikeperch (*Sander lucioperca*Linnaeus), shifts in PMRNs were rarely correlated with potential environmental variables including temperature, stock demography, year class strength, or population size, leading to the conclusion that observed phenotypic shifts in PMRNs were due primarily to fishing pressure (Kokkonen et al. 2015). Moreover, growth traits in yellow perch may be somewhat heritable. One study in a relatively small number of full-sib families estimated heritabilities between 0.075 and 0.14 for length and weight, respectively (Cao et al. 2012), while other studies have shown strong family effects and genotype by environment interactions for growth during the first 2 years of life (Wang et al. 2009a, 2011). In the closely related percid walleye (*Sander vitreus*Mitchill), heritability of length and weight ranged from 0.30 to 0.93 (Kapuscinski et al. 1996). Beyond percids, Law (2000) found evidence for heritability of several life history traits in fishes (0.24 for weight, 0.30 for length, 0.31 for age at maturation) across a number of studies. Strong genetic correlations between growth and maturity have also been observed in Atlantic cod, meaning shifts in one likely represent changes in the other trait as well (Kristjánsson and Arnason 2014). Finally, selective fishing exerts extremely strong selective pressure on fish populations—in one study, natural selection only overcame fisheries selection when fishing declined, and the two forces rarely acted in concert (Edeline et al. 2007). These lines of evidence suggest that (i) yellow perch life history traits may respond to size-selective harvest in a heritable manner, and (ii) fisheries selection likely exerts strong selective pressures on yellow perch populations.

Yellow perch is also a shorter-lived, earlier-maturing species than most of the large-bodied marine species upon which research on fisheries-induced evolution has focused in the wild (Grift et al. 2003; Barot et al. 2004b; Olsen et al. 2004; Hard et al. 2008); thus, their shorter generation time could allow yellow perch to respond more quickly than a species with a much longer generation time. Phenotypic change in response to anthropogenic disturbances appears to follow a pattern of rapid, abrupt change following the disturbance (Hendry et al. 2008), and this study covered a period of highly dynamic changes to Great Lakes ecosystems, both due to changes in fishing pressure and other stressors (Allan et al. 2013), meaning the years included in this study may have captured a phase of abrupt responses. Haldanes are also sensitive to estimation of generation time and the time interval of change (Devine et al. 2012). Yellow perch generation times encapsulated only a few generations in most cases in this study (3.4–5.1 generations from the 1980s to 2000s across populations) which may have resulted in overestimation of rates of change. Even so, we observed rates twice as large as reported elsewhere in the literature, so the conclusion that yellow perch maturation schedules have shifted extremely rapidly in response to recent system changes likely remains valid despite these caveats.

Alternatively, disruptive selection by historic commercial gill net fisheries, which primarily select for intermediately-sized fish and do not capture the smallest or largest individuals, may have increased phenotypic variance and responsiveness of yellow perch life history traits. Although gill nets were likely able to capture the majority of available individuals in a given year (Wilberg et al. 2005), it is possible that such extreme selection for the survival of either very small or very large individuals imposed a disruptive selection regime. In addition, despite providing size refuges for both large and small individuals, intense exploitation in gill net fisheries can still lead to rapid and abrupt decreases in age at maturation once fishing mortality becomes sufficiently high (Jørgensen et al. 2009). Species that naturally tend to mature earlier in life and are subject to adaptive fisheries management strategies (such as Great Lakes yellow perch) also appear to be most sensitive and susceptible to disruptive selection on life history traits, which may result in either a shift to trait dimorphism or generally increased population-level variance in life history trait expression (Landi et al. 2015). This increased variance may improve the adaptability of fish stocks to future changes in selection regimes and may improve the resilience of stocks to fisheries-induced evolution (Jørgensen et al. 2009). Increased phenotypic variance due to fisheries-induced disruptive selection was suggested to have allowed Lake Windermere pike (*Esox lucius*Linnaeus) to rapidly recover growth traits following a relaxation of fishing pressure (Edeline et al. 2007, 2009). Therefore, disruptive commercial harvest may have improved the ability of yellow perch stocks to respond to new, lower harvest rates.

**Figure 4:**
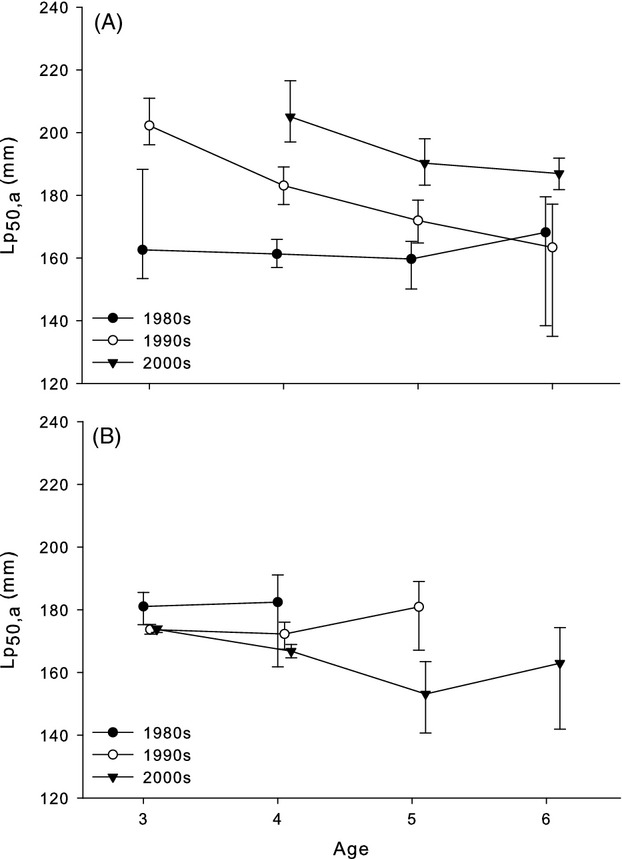
Comparison of trends in the height and slope of female yellow perch PMRNs from (A) Lake Michigan, where the commercial fishery was closed in 1997, and (B) central Lake Erie, where fishing has continued throughout the study period. Different symbols represent different decades (1980s, 1990s, and 2000s), and error bars represent 95% credible intervals of posterior distributions. Points are offset on the *x*-axis by 0.1 years for clarity.

Finally, yellow perch exhibits positive maternal effects on egg size, larval size, and larval provisioning, potentially improving survival of larvae produced by older or larger females (Heyer et al. 2001; Andree et al. 2014). Such maternal effects could enhance reproductive benefits for females that delay maturation in favor of increasing size. This is a concept that has remained largely unaccounted for in modeling studies of evolutionary recovery from fishing, even as others have suggested the slow recovery of life history traits may result from relatively little fitness benefit for delaying maturation (e.g. Law 2000; Enberg et al. 2009; Kuparinen and Hutchings 2012). The temporal shift in the Lake Michigan female PMRN from a flat slope to a strongly negative slope, while central Erie female PMRNs remained largely flat (Fig.4), may support the hypothesis that maternal effects can influence the adaptive response of populations to changes in size-selective fishing pressure (Hutchings 2004). In general, we observed largely sex-specific changes in PMRNs, where females exhibited large changes in PMRNs while males generally exhibited little change. A similar pattern was observed in several species reviewed by Devine et al. (2012)—PMRNs in males tended to exhibit less change than for females in a given stock. This could be the result of very different suites of life history trade-offs apparent for males and females, reducing the relative importance of size to reproductive success in males compared to females (Diana and Salz 1990; Collingsworth and Marschall 2011), in addition to sexually dimorphic growth patterns reducing the relative susceptibility of immature males to harvest (Wilberg et al. 2005). This evidence potentially supports the hypothesis that maternal effects on reproductive fitness may influence life history responses to harvest. As size- and age-based maternal effects have been observed in a number of exploited species (e.g. Marteinsdottir and Steinarsson 1998; Venturelli et al. 2009; Hixon et al. 2014), further examinations of the importance of maternal effects to life history adaptation could yield new predictions for the adaptive trajectories of exploited stocks.

Most research on fisheries-induced changes in maturation paints a dire picture of near-permanent genetic changes to exploited stocks, causing decreased yields and prolonged recovery on the order of centuries or millennia (Hutchings and Reynolds 2004; Walsh et al. 2006; Hard et al. 2008; Enberg et al. 2009; Devine et al. 2012; Sutter et al. 2012; Kuparinen et al. 2014). Indeed, the stocks in this study that experienced continuous strong commercial harvest showed either no recovery or even a small decline in PMRNs. However, the increases in yellow perch PMRNs following reduced harvest in Lake Michigan and, to a lesser extent, Saginaw Bay, may offer evidence that harvest-induced changes in life history traits are not necessarily as slow to recover as previously thought. In light of this study and others documenting the recovery of traits related to body size and growth (Edeline et al. 2007; Conover et al. 2009), maturation (Olsen et al. 2004), and egg size, larval size, and larval viability (Salinas et al. 2012), it now appears that some fish stocks may retain the ability to recoup previous trait distributions. Moreover, the rapidly shifting maturation schedules we have documented have potential ramifications for the ability of management plans to adequately set and meet fishing mortality targets for exploited populations (Thorson et al. 2015). These results further stress the need for regular monitoring of fish life history traits and careful consideration of the proper metrics (e.g. A_50_, PMRN) with which to estimate them. Furthermore, the rapid recovery of maturation schedules following declines in fishing or fishing moratoria indicates that rapid and extensive management actions following the detection of population declines can feasibly slow, prevent, or even reverse the effects of fishing on population vital rates. Managing for stock diversity and natural connectivity among subpopulations may also be an important consideration for stock resilience and recovery, as dispersal between subpopulations in Lake Michigan may have contributed to the rapid recovery of life history traits following the moratorium. In sum, this study joins many others in the call for a full inclusion of the principles of Darwinian fisheries management into regulatory efforts (Jørgensen et al. 2007), in combination with regular assessment of plastic and evolutionary trait changes (Kuparinen and Merilä 2007), which may provide avenues for the mitigation and prevention of fisheries-induced changes to important life history traits.

## Data archiving statement

Raw data for this study are available from the Purdue University Research Repository (PURR): http://dx.doi.org/10.4231/R73N21BS.
